# COVID-19 preparedness: capacity to manufacture vaccines, therapeutics and diagnostics in sub-Saharan Africa

**DOI:** 10.1186/s12992-021-00668-6

**Published:** 2021-03-03

**Authors:** Bisi Bright, Chinedum Peace Babalola, Nadia Adjoa Sam-Agudu, Augustine Anayochukwu Onyeaghala, Adebola Olatunji, Ufuoma Aduh, Patrick O. Sobande, Trevor A. Crowell, Yenew Kebede Tebeje, Sunny Phillip, Nicaise Ndembi, Morenike Oluwatoyin Folayan

**Affiliations:** 1 COVID-19 Think Tank, Nigeria; 2 Live Well Initiative Academy Nigeria, Lagos, Nigeria; 3 Department of Pharmaceutical Chemistry, Faculty of Pharmacy, University of Ibadan, Ibadan, Nigeria; 4 Centre for Drug Discovery, Development & Production, University of Ibadan, Ibadan, Nigeria; 5 Genetics & Bioethics Unit, Institute of Advanced Medical Research & Training, College of Medicine, Ibadan, Nigeria; 6 College of Basic Medical Sciences, Chrisland University, Abeokuta, Ogun State Nigeria; 7 Institute of Human Virology Nigeria, Abuja, Nigeria; 8 Institute of Human Virology, University of Maryland School of Medicine, Baltimore, USA; 9 Department of Paediatrics, University of Cape Coast School of Medical Sciences, Cape Coast, Ghana; 10 Unit of Clinical Chemistry, Department of Medical Laboratory Science, University College Hospital, Ibadan, Nigeria; 11 Unit of Clinical Chemistry, Department of Medical Laboratory Science, Lead City University, Ibadan, Nigeria; 12 Fort Worth Internal Medicine, Fort Worth, TX USA; 13 World Health Organisation, Asaba, Delta State Nigeria; 14 Stephen’s Pedi & Pulmonary Medicine, Fort Worth, TX USA; 15 U.S. Military HIV Research Program, Walter Reed Army Institute of Research, Silver Spring, MD USA; 16 Henry M. Jackson Foundation for the Advancement of Military Medicine, Bethesda, MD USA; 17 Africa Center for Disease Control and Prevention, African Union Commission, Addis Ababa, Ethiopia; 18 Kanazawa University, Graduate School of Medical Sciences, Kanazawa, Japan; 19 Department of Child Dental Health, Obafemi Awolowo University, Ile-Ife, Nigeria

**Keywords:** COVID-19, Vaccine, Therapeutics, Diagnostics, Manufacturing capability, Sub-Saharan Africa

## Abstract

**Objective:**

The COVID-19 pandemic is a biosecurity threat, and many resource-rich countries are stockpiling and/or making plans to secure supplies of vaccine, therapeutics, and diagnostics for their citizens. We review the products that are being investigated for the prevention, diagnosis, and treatment of COVID-19; discuss the challenges that countries in sub-Saharan Africa may face with access to COVID-19 vaccine, therapeutics, and diagnostics due to the limited capacity to manufacture them in Africa; and make recommendations on actions to mitigate these challenges and ensure health security in sub-Saharan Africa during this unprecedented pandemic and future public-health crises.

**Main body:**

Sub-Saharan Africa will not be self-reliant for COVID-19 vaccines when they are developed. It can, however, take advantage of existing initiatives aimed at supporting COVID-19 vaccine access to resource-limited settings such as partnership with AstraZeneca, the Coalition for Epidemic Preparedness and Innovation, the Global Alliance for Vaccine and Immunisation, the Serum Institute of India, and the World Health Organization’s COVID-19 Technology Access Pool. Accessing effective COVID-19 therapeutics will also be a major challenge for countries in sub-Saharan Africa, as production of therapeutics is frequently geared towards profitable Western markets and is ill-adapted to sub-Saharan Africa realities. The region can benefit from pooled procurement of COVID-19 therapy by the Africa Centres for Disease Control and Prevention in partnership with the African Union. If the use of convalescent plasma for the treatment of patients who are severely ill is found to be effective, access to the product will be minimally challenging since the region has a pool of recovered patients and human resources that can man supportive laboratories. The region also needs to drive the local development of rapid-test kits and other diagnostics for COVID-19.

**Conclusion:**

Access to vaccines, therapeutics, and diagnostics for COVID-19 will be a challenge for sub-Saharan Africans. This challenge should be confronted by collaborating with vaccine developers; pooled procurement of COVID-19 therapeutics; and local development of testing and diagnostic materials. The COVID-19 pandemic should be a wake-up call for sub-Saharan Africa to build vaccines, therapeutics, and diagnostics manufacturing capacity as one of the resources needed to address public-health crises.

## Background

Coronavirus disease 2019 (COVID-19) is a viral infection caused by severe acute respiratory syndrome coronavirus 2 (SARS-CoV-2). By the end of November 2020,, there were over 62 million cases of COVID-19 and 1.4 million deaths (case fatality rate 2.4%) had been reported globally [1]. At this time also, Africa had recorded over 2 million COVID-19 cases and over 49,000 [1]. Resource limitations in sub-Saharan Africa contribute to relatively lower access to COVID-19 diagnostic testing, which result in underdiagnosis, especially of asymptomatic or pauci-symptomatic persons, and limited basic and advanced care to prevent morbidity and mortality [2–5]. Notwithstanding, COVID-19 has overwhelmed even highly developed healthcare systems in Europe and the Americas [6–9].

In sub-Saharan Africa, proactive public-health interventions and thoughtful allocation of resources will be needed to prevent poor patient outcomes and control the pandemic. The most widely adopted control measures for COVID-19 are behaviour change and physical/social distancing, such as closures of schools and/or workplaces, reduction of public and religious gatherings, hand hygiene, respiratory hygiene, proper use of face masks in public places, internal travel restrictions, and border closures [5, 10]. Guidelines typically require persons diagnosed with COVID-19 to be isolated for a minimum of 10 days (this may be extended for as long as there is no evidence of viral clearance), and 14-day quarantine of their contacts, either in their homes or in dedicated facilities [11]. If appropriately implemented, these measures can reduce disease transmission and slow the spread of SARS-CoV-2 between and within countries. However, eradication of COVID-19 will likely require a safe and effective vaccine to prevent disease acquisition independent of individual behaviours [12]. As of 5th November 2020, there were 104 active studies of candidate COVID-19 vaccines worldwide, registered in Clinicaltrials.gov [13]. Thirteen (12.5%) of these studies are being conducted in Africa, with 11 (10.6%) in sub-Saharan Africa (Table 1). Candidate vaccines in the United Kingdom [14], the United States [15] and China [16] have demonstrated safety and immunogenicity in Phase I studies, thus providing early and reassuring signals that an effective COVID-19 vaccine is in progress.

**Table 1:** Registered COVID-19 Therapeutics and Vaccine Trials in Sub-Saharan Africa as of November 5, 2020

Country	Title	Interventions	Sponsor/Collaborators	Phase	Enrolment target	Enrolling Children < 18 years	Start date
**Ghana**	**PROTECT-Surg** Preventing Pulmonary Complications in Surgical Patients at Risk of COVID-19	Drug: Hydroxychloroquine Drug: Lopinavir/Ritonavir Drug: Hydroxychloroquine + Lopinavir/Ritonavir Drug: standard of care	National Institute of Health Research, UK | University for Development Studies School of Medicine and Health Sciences Tamale Teaching Hospital	Phase 3	6400	Yes (≥16 years)	25-Apr-20
**Kenya** **South Africa**	A study to determine if a new COVID-19 vaccine safely generates protective immune responses in adults in Kenya	Single dose of ChAdOx1 nCoV-19 vaccine vs Verorab (rabies vaccine)	University of Oxford KEMRI Wellcome Trust Program University of the Witwatersrand, Johannesburg	Phase 1/2	400 in Kenya 2000 (1950 HIV-uninfected and 50 people living with HIV) in South Africa	No	28-Oct-20 in Kenya 24-Jun-20 in South Africa
**Nigeria**	**LACCTT** Lagos COVID-19 Chloroquine Treatment Trial	Drug: Chloroquine phosphate Drug: Hydroxychloroquine sulphate	Lagos State Government Nigerian Institute of Medical Research	Not Applicable	600	No	17-Apr-20
	**CRASH-19** Coronavirus Response - Active Support for Hospitalised COVID-19 Patients	Drug: Aspirin Drug: Losartan Drug: Simvastatin	London School of Hygiene and Tropical Medicine	Phase 3	1000	No	01-Apr-20
	**IHP Detox tea trial** Efficacy and safety of IHP Detox Tea for treatment of Corona virus disease 2019: a pilot placebo-controlled randomized trial	IHP Detox Tea (a special blend of *Andrographis paniculata*, *Garcinia kola* and *Psidium guajava*)	Neimeth International Pharmaceuticals Plc	Not Applicable	72	No	01-May-20
	**IVERCOVID** Does ivermectin cure and/or prevent COVID-19?	Drug: ivermectin 6 mg Drug: ivermectin 12 mg Drug: placebo	Rachel Eye Center, Lagos University Teaching Hospital	Phase 3	45	Yes (stated as ‘all ages’)	23-Apr-20
**Senegal**	**SEN-CoV-Fadj** Efficacy and Safety Evaluation of Treatment Regimens in Adult COVID-19 Patients in Senegal	Drug: Hydroxychloroquine Drug: Hydroxychloroquine + Azithromycin	Institut Pasteur de Dakar Fann Hospital, Senegal Ministry of Health, Senegal Diamniadio Children Hospital, Senegal Dalal Jamm Hospital, Senegal Epicentre, Paris, France	Phase 3	258	Yes (≥15 years if married; ≥18 years if not married)	01-Jun-20
	**ESHAZ Trial** Hydroxychloroquine, Azithromycin and Zinc for the treatment of SARS-CoV-2 infection in Senegal	Drug: Hydrochloroquine 600 mg plus Azythromycin Drug: Hydrochloroquine 400 mg plus Azythromycin Drug: Zinc	Faculty of Medicine University Cheikh Anta Diop of Dakar Senegal.	Phase 3	384	No	01-Jun-20
**South Africa**	**CQOTE** Chloroquine Outpatient Treatment Evaluation for HIV-COVID-19	Drug: Chloroquine or hydroxychloroquine	University of Cape Town	Phase 3	560	No	01-May-20
	BCG Vaccination for Healthcare Workers in COVID-19 Pandemic	Biological: Bacille Calmette-Guerin (BCG) Other: Placebo Comparator	TASK Applied Science (University of Cape Town)	Phase 3	500	No	04-May-20
**Sudan**	**GA & COVID19** Potential Role of Gum Arabic as Immunomodulatory Agent Among COVID 19 Patients	Dietary Supplement: *Acacia Senegal* Dietary Supplement: Pectin	Al-Neelain University University of Khartoum	Phase 2 Phase 3	110	Yes (≥5 to 90 years)	01-Jun-20
**Kenya** **South Africa**	**SOLIDARITY** Public health emergency SOLIDARITY trial of treatments for COVID-19 infection in hospitalized patients	1. Local standard of care alone OR local standard of care plus one of 2. Remdesivir 3. Chloroquine or hydroxychloroquine 4. Lopinavir + ritonavir 5. Lopinavir + ritonavir plus interferon-beta	World Health Organization	Phase 3	10,000	No	01-Mar-20
**Cameroon** **Ghana** **Uganda** **South Africa** **Zambia** **Zimbabwe**	**CROWN CORONATION:** Chloroquine RepurpOsing to healthWorkers for Novel CORONAvirus mitigaTION	Drug: Low-dose chloroquine/hydroxychloroquine Drug: Mid dose chloroquine or hydroxychloroquine Drug: High dose chloroquine or hydroxychloroquine Drug: Placebo	Washington University School of Medicine/Bill and Melinda Gates Foundation	Phase 3	55,000	No	01-Apr-20
**Madagascar**	Efficacy of New COVID 19 Treatment	Efficacy of Artesunate IV alone or combined with vitamin C IV for the treatment of COVID-19	Ministry of Health, Madagascar	Phase 2		No	29-Apr-20
**South Africa**	**ENSEMBLE** Ad26.COV2.S1 for the Prevention of SARS-CoV-2-Mediated COVID-19 in Adult Participants	Biological: Ad26.COV2.S1 Other: Placebo	Janssen Pharmaceutical Companies University of the Witwatersrand, Johannesburg	Phase 1/2	6000 from Argentina, Brazil, Chile, Columbia, Mexico, Peru, Philippines, USA, Ukraine and South Africa	No	2-Nov-20
**South Africa**	Safety, Tolerability, Immunogenicity, and Efficacy of RNA Vaccine Candidates Against COVID-19 in Healthy Individuals	Biological: BNT162b1 Biological: BNT162b2 Other: Placebo	Pfizer and Biontech	Phase 2/3	11,000 from U.S., Argentina and Brazil, Germany, Turkey and South Africa	No	No date
**South Africa**	Safety and Immunogenicity of a SARS-CoV-2 rS Nanoparticle Vaccine With/Without Matrix-M Adjuvant	NVX-CoV2373	Novavax Coalition for Epidemic Preparedness Innovations University of the Witwatersrand, Johannesburg	Phase 2	2904 (2650 HIV-uninfected and 240 people living with HIV)	No	17-Aug-20

Search strategy: The following clinical trials registers were searched: clinicaltrials.gov, covid-trials.org, the Pan African Clinical Trial Registry (PACTR) and WHO international clinical trials registry platform (ICTRP). The following search terms were used: “COVID” (subject field) AND “interventional” OR “Randomized” (study type). Studies conducted in all 55 African Union member states are listed after cross checking for duplicates across registries. Multi-centric trials are listed as one entry

Unfortunately, vaccine research and development are lengthy processes that typically require years to complete. Pharmacologic agents that have demonstrated safety for other diseases may be a useful bridge for preventing or treating COVID-19 while an effective vaccine is being developed. Hydroxychloroquine showed promise for pre-exposure prophylaxis against COVID-19 from an observational study pre-print among healthcare workers [17], however it was reported ineffective for post-exposure prophylaxis in this population in another study [18]. There are also published COVID-19 protocols of trials of hydroxychloroquine or chloroquine and melatonin as pre-exposure prophylaxis for healthcare workers in Spain [19], but no trials for post- and pre-exposure prophylaxis in sub-Saharan Africa have been as yet registered (Table 1).

For most of the pandemic so far, COVID-19 therapy has focused on symptom control and supportive care. Remdesivir was approved for compassionate use by the United States Food and Drug Administration after a clinical trial showed shorter duration of hospitalization [20] and improved clinical outcomes in hospitalized patients who received the drug [21]. However, other studies, including from China [22] have found only marginal benefits. Recently, preliminary preprint results of the worldwide WHO Solidarity trial among 11,266 adults at 405 hospitals in 30 countries indicated that remdesivir had no measurable benefit in mortality or disease course [23]. Compassionate use of humanized Virus Suppressing Factor-variant 13 (hzVSF-v13) in two Korean patients with severe COVID-19 has also shown promising results [23]. Finally, in a large randomized trial, dexamethasone was found to reduce the risk of death from 41 to 28% inpatients on mechanical ventilators, and reduced the risk of death among patients receiving oxygen from 25 to 20% [24]. As of 5th November 2020, there were 22 drug/therapeutics trials being conducted in 11 countries across sub-Saharan Africa (Table 1).

In anticipation of favourable outcomes, plans are being put in place for mass production of effective vaccines and therapeutics. The COVID-19 Therapeutics Accelerator, funded by Bill and Melinda Gates; the Wellcome Trust; and the Mastercard Impact Fund plan to work with regulators to fast-track drug approval processes and rapidly manufacture and distribute effective interventions to patients [25]. Mass production of standardized serological assays to detect functional antibodies in different stages and severity of disease, including indicating the presence of protective antibodies will also be needed [26].

In this article, we review products under investigation for the prevention, diagnosis, and treatment of COVID-19. We discuss the challenges that countries in sub-Saharan Africa may face with access to COVID-19 vaccines, therapeutics, and diagnostics due to the limited manufacturing capacity on the continent. Lastly, we make recommendations on actions to mitigate these challenges and ensure health security in sub-Saharan Africa during this unprecedented pandemic. The discussion will also be important for preparedness for COVID-19 as well as for future public-health crises.

### Vaccines to prevent SARS-CoV-2 infection and COVID-19

Several COVID-19 vaccine concepts are being tested worldwide, but only five candidate vaccines are being tested in sub-Saharan Africa (Table 1). These include Bacille Calmette-Guérin (BCG) vaccine, historically used for prevention of tuberculosis, and is being evaluated for the prevention of COVID-19 among healthcare workers in South Africa [25]; similar studies are being conducted in Australia, Netherlands, the United States, France and Germany [27–30]. Testing of BCG is felt justified based on its effectiveness in reducing deaths from tuberculosis pneumonia and sepsis-related deaths in neonates, and in reducing respiratory tract infections in adolescents [31, 32]. The mechanism of action against SARS-CoV-2 is not known, but it is theorized that the BCG vaccine might reduce viraemia after SARS-COV-2 exposure, thereby reducing the risk of severe COVID-19 and rapid recovery [30]. Others however counter-hypothesize that provocative epidemiological associations between BCG and better COVID-19 outcomes are a correlation and not causation,, in that countries with high coverage for mandatory vaccinations have strong public health systems that are robustly responding to the COVID-19 pandemic [33]. Furthermore people in countries with have high uptake for these mandatory vaccinations are likely to be more risk averse and comply with pandemic control measures [33].

There is also the ChAdOx1 nCoV-19 vaccine trial in South Africa that is recruiting adults with and without HIV infection [34]. This study is conducted in collaboration with the Oxford University and the Jenner Institute, and the same study is also planned for implementation in Kenya [35]. NVX-CoV2373 is a vaccine product of Novavax, a US drug developer of next-generation vaccines for serious infectious diseases, and is being tested in South Africa, in addition to the Ad26.COV2-S, a Johnson & Johnson product [36]. There are also plans for COVID-19 vaccine trials underway in Uganda in collaboration with the Imperial College London [36].

Although only five discrete vaccine trials are ongoing/planned for implementation in several countries, sub-Saharan Africa has the human and institutional resources required to host and participate in many more COVID-19 trials, based on current and past experiences with HIV, Ebola, malaria, and tuberculosis vaccine trials [37–43]. The region also has human-vaccine production plants in Senegal (prequalified yellow fever vaccine) and South Africa (fill-finish) [44, 45]. Although no country in sub-Saharan Africa produces BCG vaccines, those countries with fill-finish vaccine may be able to negotiate with vaccine manufacturers to produce COVID-19 vaccines to meet the region’s need, if said vaccines are found effective..

### Pharmacologic interventions to prevent and treat COVID-19

Medications for pre- and post-exposure prophylaxis against other infectious diseases such as HIV, have been proven to curb the spread of infections in key populations. Also, the prompt identification and treatment of patients with COVID-19 will decrease morbidity, mortality, and transmission of the SARS-CoV-2 virus. Several products, re-purposed after being investigated or licensed for use in other infectious diseases, are being investigated for potential roles in prophylaxis and therapy for COVID-19. These products act by five mechanisms: inhibition of virus replication, inhibition of entry of the virus into cells, killing the virus, augmenting a physiologic immune response, or treating symptoms. The drugs can be classified into antivirals, anti-parasitics, antibiotics, and immunomodulators.

#### Antivirals

Major antivirals that have in vitro activity against SARS-CoV-2, have undergone or are undergoing investigation, and/or are approved for compassionate use are: lopinavir/ritonavir [46], favipiravir [47], umifenovir [48], and remdesivir [21, 46, 49]. Lopinavir/ritonavir is a boosted protease inhibitor widely used for treatment of HIV in low- and middle-income countries and especially in sub-Saharan Africa. Early evidence suggested that lopinavir/ritonavir has little to no effect in the treatment of COVID-19 [50, 51], and the World Health Organisation Solidarity trial also found no effect of lopinavir alone and with interferon-beta 1a [23]. However, recent studies report provocative findings regarding possible benefits of lopinavir/ritonavir in combination with ribavirin and interferon-beta 1b in mild to moderate disease [52]. Favipiravir is an RNA polymerase inhibitor used for the treatment of novel strains of influenza in Japan [53].. Umifenovir (also known as arbidol) is a viral fusion inhibitor used for treatment of influenza in Russia and China, and in a recent Chinese study (in preprint), patients had inferior clinical recovery rate and relief from COVID-19-related fever and cough compared to favipiravir [54].

To date, remdesivir, an RNA viral polymerase inhibitor, has been the most promising antiviral against COVID-19. In a large randomized trial, it shortened length of hospitalization and improved clinical outcomes among patients who required mechanical ventilation [21]. Remdesivir is manufactured in the United States and has been approved for compassionate use for treatment of severe COVID-19 in that country, and in Japan, Singapore, and India [55–58]. The drug is given by intravenous infusion for five to 10 days, and costs approximately £2000 [€2205; $2600] per course [59], although the manufacturer Gilead has announced that the cost of a five day course will likely drop [60]. The global WHO Solidarity trial however showed no impact or trend for remdesivir towards improving 28-day mortality among COVID-19 patients [23, 59].

#### Antiparasitics

The main anti-parasitics of interest for COVID-19 prevention and treatment are chloroquine and hydroxychloroquine, which have been used as anti-malarial drugs in low- and middle-income countries for nearly 80 years. While both antiparasitics have in vitro activity against SARS-CoV-2, hydroxychloroquine is a more potent [61] and less toxic derivative of chloroquine [62]. Metanalyses and large trials evaluating chloroquine and/or hydroxychloroquine for COVID-19 treatment (including the WHO Solidarity trial) have showed no efficacy in reducing mortality, need for mechanical ventilation, or duration of hospital stay [23, 63–65]. There are still ongoing trials for chloroquine/hydroxychloroquine pre-exposure and post-exposure prophylaxis, globally, including in Africa [61, 66]. Although the two drugs are widely available in sub-Saharan Africa because of their utility for treatment of malaria treatment, caution has been raised over their indiscriminate use for treatment of COVID-19 outside experimental or medically guided prescription [62].

More than 12 major manufacturers in Asia Pacific, Europe, North Africa, the Middle East and the Americas supply the global hydroxychloroquine market, but there are currently no manufacturers of the agent in sub-Saharan Africa [67]. India, the world’s largest producer of hydroxychloroquine [67], initially banned exporting hydroxychloroquine to protect domestic demand in the wake of the pandemic, but giving emerging trial data on little impact of the drug in COVID-19 treatment, it lifted the ban in June 2020 [68]. Emerging observational, non-randomized studies from African countries also indicate either no treatment or mortality effect [69], or possibly, a shortening of hospital stay compared to azithromycin or no treatment [70]. Regardless, access to hydroxychloroquine will be a challenge for sub-Saharan African countries if evidence should support its use in preventing COVID-19. Countries in the region are rising to the challenge, however: Cipla, a pharmaceutical company in Uganda announced its plan to start producing hydroxychloroquine in May 2020 [71]. The anticipated cost to patients is just $1 per 14-day course, or approximately $0.07 per day [72].

Also of interest is ivermectin, an antiparasitic used for the treatment of onchocerciasis, among other parasitic infections. It has in vitro efficacy against SARS-CoV-2 replication [ref], like it has against a broad spectrum of other RNA viruses [73, 74]. It is yet to be evaluated in a well-designed randomized control trial for its effectiveness in COVID-19 treatment, though the human trial dose required is 10,000 times larger than that used in cell culture, which makes it less likely to serve as a candidate for COVID-19 clinical trials [75]. However, if found effective at safe doses, the cost may not be prohibitive to African countries, as it is has been made available for free by manufacturer Merck & Co. Inc. since 1987, and for as long as it was needed [76].

#### Antibiotics

Antibiotics used in the management of COVID-19 have been recommended specifically for treatment of suspected or confirmed bacterial superinfection [77]. However, macrolides such as azithromycin have been proposed to be used either alone or in combination with other drugs (eg hydroxychloroquine/chloroquine) for the primary treatment of COVID-19. However, there is still insufficient clinical trial evidence to substantiate this clinical application [78]. In in vitro studies, azithromycin has not been effective against SARS-CoV-2, but when combined with hydroxychloroquine, it inhibited viral replication [79]. Azithromycin may also be beneficial through its broad anti-inflammatory properties [80]. Evaluation of the effectiveness of azithromycin in clinical COVID-19 studies has been hampered by small numbers of study participants, methodological flaws and concerns about cardiotoxicity, especially in combination with chloroquine/hydroxychloroquine [65, 81, 82]. As such, there is insufficient clinical evidence for the use of azithromycin alone or in combination, in the treatment of COVID-19 [78, 80].

#### Immunomodulators

Associations between high expression of interleukin (IL)-1 andIL-6, and severe COVID-19 have been reported [83, 84]. Interferon beta-1a and inhibitors of IL-1 (anakinra) and IL-6 (sarilumab, siltuximab, tocilizumab) have been used as adjunct treatments for severe COVID-19 to ameliorate tissue damage caused by pro-inflammatory cytokines [85, 86]. Observational/cohortstudies using IL-1 inhibitor anakinra [87, 88] and IL-6 inhibitor tocilizumab [89–92] have reported benefits in clinical improvements and survival. Clinical trials evaluating IL-1 and IL-6 inhibitors in COVID-19 are ongoing [93–95]. Results from the WHO Solidarity trial indicate that interferon beta-1a is not efficacious in reducing COVID-19 mortality [23]. The costs of immunomodulator infusions range from approximately $50 (Canadian dollars) per day for 100 mg of anakinra, and up to CAD $900 per 400 mg dose of tocilizumab [96]. Anakinra, tocilizumab and similar immunomodulatory agents are produced largely by Sweden Orphan Biovitrum, Regeneron Pharmaceuticals, EUSA Pharma, and Roche—all located in the global North [97–100].

The use of convalescent plasma (which is antibody-rich plasma from individuals who have recovered from a specific infection) and hyperimmune immunoglobulin (concentrated preparation of highly antibody-rich immunoglobulin) have been impactful in the treatment of severe viral respiratory diseases. COVID-19- specific convalescent plasma and hyperimmune globulin have been evaluated for treatment of SARS-CoV-2 infection in observational studies [101, 102] and randomized trials [103, 104]. While some individual studies report benefits [101, 105, 106], an Indian trial [104] and multi-study analyses [102, 103, 107] have been inconclusive with regard to impact on clinical improvement and mortality; the analyses cite small cohorts, weak/poor study design (including poor comparability of co-interventions) as contributing factors. Clearly, more data from rigorously-designed and large randomized trials are needed, and some of these are under way [108–110]. If found conclusively effective, convalescent plasma and hyperimmune globulin may be have good prospects for use in Africa due to the relative ease of preparation and storage of hyperimmune immunoglobulin from COVID-19 convalescent blood [111]. Even so, high costs of these treatments will be a potential barrier to access and scale-up.

#### Traditional medicines and African compounds

Amidst the flurry of new and repurposed drugs under investigation against SARS-CoV-2 infection, locally developed or locally obtained compounds from African countries have been presented as possible COVID-19 therapeutics. The Madagascar herbal tonic (branded Covid-Organics), developed by the Malagasy Institute of Applied Research, is said to contain *Artemisia annua*, *Cinnamomum camphora,* essential oils, flavonoids, coumarins, polysaccharides, saponins, tannins, and pentacyclic triterpenes has been promoted as a cure for COVID-19 [112, 113], but it has not been tested against SARS-CoV-2 in vitro or in vivo. The World Health Organisation and Africa Centers for Disease Control have cautioned the public against the use of this as-yet unproven tonic and other untested herbal remedies [114, 115], however these two entities have convened a panel of experts who have developed a protocol and terms of reference for COVID-19 herbal medicine clinical trials [116] A recent preprint reports in vitro activity of *Artemisia annua* against SARS-CoV-2 [117], and the product has been listed for clinical testing, as reflected in Table 1.

NIPRIMUNE was developed as an immunostimulant by the National Institute for Pharmaceutical Research and Development Nigeria, and unveiled in 2018 as an adjunct treatment for HIV [118]. It is reported to have with potential as adjunct therapy for COVID-19 [119], but there is currently no clinical trial data available on its effectiveness against SARS-CoV-2 nor HIV.. In Ghana, the Center of Awareness CoA mixture (formerly CoA FS), is reported to contain 160 phytochemicals, and is commercially available as an immune booster supplement targeted for use by people living with HIV [120]. It is also being touted as immune support and treatment for COVID-19 symptoms and is reported to be undergoing safety and anti-viral efficacy studies at Ghana’s Centre for Scientific Research into Plant Medicine [121]. A 250 ml bottle of CoA mixture sells for approximately $21; dosing is 10–20 mls twice daily [120]. There is currently no clinical trial data on the effectiveness of CoA mixture against HIV or SARS-CoV-2. No related studies are registered in ClinicalTrials.gov, as reflected in Table 1.

#### Other therapies

Corticosteroids have historically not been recommended for treatment of viral pneumonias such as those caused by respiratory syncytial virus, influenza virus, the 2003 Severe Acute Respiratory Syndrome coronavirus, and Middle Eastern Respiratory Syndrome coronavirus,because of their lack of clinical effectiveness and risk of adverse effects [122, 123]. However, the United Kingdom’s RECOVERY Trial found dexamethasone able to significantly reduce 28-day mortality among COVID-19 patients; by 18% among those requiring oxygen and by 36% for those on mechanical ventilation [24]. The lowest cost of a4 mg/ml injectable solution of dexamethasone is ~$25 for 25 ml [124]. The 20 companies that supply dexamethasone globally are in seven countries (China, India, United States, Spain, Germany, France, Malaysia), none in sub-Saharan Africa [125].

The potential impact of dietary supplements such as vitamin C and zinc, in decreasing the duration of COVID-19 symptoms and prevent disease progression in the ambulatory setting is being investigated. Additionally, on the heels of provocative findings in some observational studies [126–128], anticoagulant and antiplatelet therapies are being studied for use in patients with COVID-19 with rapidly deteriorating pulmonary, cardiac, or neurological function, or with sudden, localized loss of peripheral perfusion [129, 130].

### COVID-19 diagnostics

Robust diagnostic testing to differentiate SARS-CoV-2 from other viral agents causing respiratory infections is needed. As a public health response, millions of cases and contacts must be tested in a timely manner to break the chain of transmission. The viral nucleic acid real-time reverse transcription-polymerase chain reaction (rRT-PCR) assay is the standard for the molecular diagnosis of COVID-19 [131]. Effective management of COVID-19 across countries is therefore dependent on a consistent supply of test kits and laboratory capacity to utilize them [132]. The immense demand for COVID-19 test kits has led to a global shortage, which has been worse in Africa, where countries who may have funds cannot procure the products because of supply shortages [133]. Pricing for COVID-19 testing varies widely globally, and even within countries, and thus these price variations will have to be better regulated as testing is scaled up beyond studies and state-level surveillance.

In response to the rapidly growing need for and the shortage of rRT-PCR assay kits, many diagnostic-test manufacturers are developing and supplying rapid-test kits to detect SARS-CoV-2 [134]. Rapid SARS-CoV-2 antigen detection tests can detect active infection, and antibody testing complements rtPCR, at about 10 or more days or more after symptom onset, in assessing past infections [135]. Immune-based assays for detecting human IgA, IgM and/or IgG antibodies are also available [135]. Fortunately, COVID-19 diagnostics manufacturers include local African companies in Kenya, Morocco, Senegal, and South Africa [133]. Products from local manufacturers will help advance he Africa Centres for Disease Control and Prevention Partnership to Accelerate COVID-19 Testing (PACT): Test, Trace, Treat [136]. This initiative is mobilizing experts, community workers, suppliers, and other resources to test, trace, and treat COVID-19 in timely fashion, to minimize the impact of the pandemic in Africa.

## Discussion

While the direct health impact of COVID-19 has not been as dire as expected [137, 138], sub-Saharan Africa is not yet adequately pandemic-prepared. Significant gaps exist in the pipeline and in access to critical commodities such as vaccines, therapeutics, and diagnostics. At the time of this publication, the World Health Organization has not declared an effective treatment for COVID-19, although dexamethasone is currently the only therapy that has shown conclusive benefits for the management of moderate to severe disease among hospitalised patients [23]. When effective drugs or regimens are identified for the management of mild, moderate and severe COVID-19, high-income countries may stockpile them for use by their citizens, while African countries will likely have difficulty procuring these commodities, similar to the current difficulty in procuring diagnostics. The investments of South Africa in hosting trials – seven of the 18 vaccine trials in Africa – is noteworthy and a reflection of their leadership in HIV vaccine development efforts on the continent.

### Access to vaccines

If proactive measures are not undertaken, sub-Saharan Africa may not be self-reliant for COVID-19 vaccines when they are developed. At this time, European and North American countries already have agreements with AstraZeneca to ensure that they have priority access to the University of Oxford COVID-19 vaccine under development for its citizens, if the vaccine is found to be effective [139, 140]. It is encouraging, though, that partnerships between AstraZeneca, the Coalition for Epidemic Preparedness and Innovation, the Global Alliance for Vaccine and Immunisation, and the Serum Institute of India may facilitate equitable access to the Oxford vaccine through the COVID-19 Vaccine Global Access (COVAX) initiative [141]: COVAX aims to incentivize industry to make products available to all, regardless of ability to pay. The People’s Vaccine campaign [142] and the World Health Organisation’s COVID-19 Technology Access Pool [143] also are instruments that can increase access to vaccines in sub-Saharan Africa.

Sub-Saharan Africa has opportunities to address its low vaccine manufacturing capacity through the 2011–2020 Global Vaccine Action Plan, which provides universal access to critical vaccines through multiple approaches, including manufacturing [144]. The Plan is a roadmap for achieving the vision of the Decade of Vaccines through the transfer of vaccine manufacturing technology to African countries to enable them to meet their vaccine needs, including for emerging infectious diseases. For example, a call for implementation of the Plan was made at the 2015 World Health Assembly shortly after the 2014 Ebola outbreak in West Africa [144].

Preceding the Global Vaccine Action Plan, there was the Developing Countries Vaccine Manufacturers Network, established in 2000 to increase the production of high-quality vaccines; South Africa is the only member country from sub-Saharan Africa [145]. There is also the African Vaccine Manufacturing Initiative, established in 2010 to promote sustainable human-vaccine manufacturing capacity in Africa [146], and has the potential to be harnessed for the COVID-19 response. Unfortunately, these initiatives have been slow in galvanising support for implementation of their mandates. However, the Coalition for Epidemic Preparedness Innovations, a global initiative established in 2017 to accelerate vaccine development against emerging infectious diseases, has been raising funds to support COVID-19 vaccine research [147]. Like the COVID-19 Therapeutics Accelerator, the Coalition could facilitate access of countries in sub-Saharan Africa to COVID-19 vaccines developed elsewhere. If not already in process, it would be opportune for the Africa Centres for Disease Control and Prevention, in partnership with the African Union, have discussions with the Coalition to negotiate access.

### Access to therapeutics

Historically, access to new and effective therapeutics has been a major challenge for African countries. Global production of therapeutics is frequently geared towards profitable Western markets and ill-adapted to African realities [148]. Africa accounts for only 3% of global medicinal drug manufacturing [149], and between 70 and 90% of the medicines consumed in sub-Saharan African countries are imported [148]. For the entire continent, South Africa and Morocco are the exception, where 70 to 80% of pharmaceutical needs are met locally [149]. Only recently has Nigeria, the most populous African country, started addressing medicine access by providing loans to indigenous pharmaceutical companies and other organizations to support development of the pharmaceutical manufacturing sector, including pharmaceutical research and development [150].

Three factors that influence the access to medicines are availability, quality, and affordability. Improving availability of COVID-19 medicines in sub-Saharan Africa will depend on the sourcing and/or local manufacture of active pharmaceutical ingredients, most of which are manufactured in China or India [151]. Dexamethasone, so far the only active pharmaceutical ingredient conclusively proven effective for the treatment of moderate to severe COVID-19, is not manufactured in Africa. Quality medicines are also needed for interruption of COVID-19 transmission and mitigation of its morbidity and mortality. External sourcing also affects affordability and access, due to the costs and logistical hurdles of importation. Thus, barriers to access to finished pharmaceutical products needed for COVID-19 management must be reduced in sub-Saharan Africa, while the capacity for local production of medicines and diagnostics is expanded. In the short term, affordability, through better pricing, can be improved by pooling procurement of pharmaceutical products by the Africa Centers for Disease Control and Prevention in partnership with the African Union.

For long-term drug manufacturing in Africa, a harmonized regulatory system is needed to address the needs of member states. The African Medicines Regulatory Harmonization established by the New Partnership for Africa’s Development is a comprehensive plan for regional regulatory approval for drugs on the continent, like that of the European Union [152]; it provides leadership in creating and enabling a favourable regulatory environment for pharmaceutical-sector development. Furthermore, countries and sub-regions can adopt the pharma parks approach, where indigenous pharmaceutical companies collaborate to build the infrastructure necessary for manufacture of both active pharmaceutical ingredients and finished pharmaceutical products, thereby driving down the cost of production and ultimately enhancing access as is being done in India [153–155]. Also, support for development of industrial agricultural parks as source of raw materials such as pharmaceutical grade starch, sugar and ethanol for the pharma industries as in Ethiopia, is another way forward [156].

If clinical trial results support the deployment of new therapies, the continent may need to negotiate access to preventive or therapeutic COVID-19 interventions. Of note, South Africa and India have submitted a proposal to the World Trade Organization to consider waiving some intellectual property rules to allow for expedited access to COVID-19 vaccines and therapeutics [157]. Finally, it is imperative that negotiations, activities and studies to scale-up therapeutics and vaccines do not exclude pregnant women, children and youth, important subpopulations in the pandemic response in sub-Saharan Africa [158, 159].

### Access to diagnostics

There is also need to drive local development of rapid- test kits and other diagnostics for COVID-19. The infrastructure developed/strengthened for the COVID-19 response can additionally be harnessed for the manufacture of drugs, vaccines, and diagnostics needed for management and control of other diseases in sub-Saharan Africa. The COVID-19 Therapeutic Accelerator, which is committed to ensuring that COVID-19 products are accessible to people in low-resource settings [160], also can assist in controlling costs. The collaboration between Africa CDC and the Foundation for Innovative New Diagnostics to build COVID-19 rapid diagnostic test capacity may fast-track the process of institutionalising diagnostics development capacity process using a South-South collaborative approach [161].

## Conclusion

Several efforts are underway to discover, repurpose, and/or develop preventive and treatment options for COVID-19. Sub-Saharan Africa’s pharmaceutical industry is poorly developed and will not be able to expediently meet the region’s prevention and treatment needs for the biomedical management of COVID-19. Lessons already learned from the pandemic underscore the need for the region to look inward and devote resources for the design, discovery, and deployment of vaccines, drugs, and diagnostic tests to meet current and future needs. This capacity- and infrastructure-building will be a crucial part of the response to the evolving COVID-19 pandemic and can be the base for effective and timely responses to future disease outbreaks.

## Data Availability

The data used and analysed are all included in the manuscript.

## Abbreviations

ARDS: Acute respiratory distress syndrome
COVID-19: Novel coronavirus disease 2019
CQ: Chloroquine
HCQ: Hydroxychloroquine
ICU: Intensive care unit
RR: Relative risk
SARS-CoV-2: Severe acute respiratory syndrome coronavirus 2
